# Recent Advances in Applications of Acidophilic Fungi to Produce Chemicals

**DOI:** 10.3390/molecules24040786

**Published:** 2019-02-22

**Authors:** Rehman Javaid, Aqsa Sabir, Nadeem Sheikh, Muhammad Ferhan

**Affiliations:** 1 Lignin Valorization & Nanomaterials Lab, Centre for Applied Molecular Biology (CAMB), University of the Punjab, 87-West Canal Bank Road, Thokar Niaz Baig, 53700 Lahore, Pakistan; rehman.camb.pu@gmail.com (R.J.); aqsa.mughal411@gmail.com (A.S.); s_nadeem77@yahoo.com (N.S.); 2 Cell and Molecular Biology Lab, Department of Zoology, University of the Punjab Quaid-e Azam Campus, 54590 Lahore, Pakistan

**Keywords:** lignocellulosic biomass, laccases, peroxidases, green biochemicals, acidophilic microbes

## Abstract

Processing of fossil fuels is the major environmental issue today. Biomass utilization for the production of chemicals presents an alternative to simple energy generation by burning. Lignocellulosic biomass (cellulose, hemicellulose and lignin) is abundant and has been used for variety of purposes. Among them, lignin polymer having phenyl-propanoid subunits linked together either through C-C bonds or ether linkages can produce chemicals. It can be depolymerized by fungi using their enzyme machinery (laccases and peroxidases). Both acetic acid and formic acid production by certain fungi contribute significantly to lignin depolymerization. Fungal natural organic acids production is thought to have many key roles in nature depending upon the type of fungi producing them. Biological conversion of lignocellulosic biomass is beneficial over physiochemical processes. Laccases, copper containing proteins oxidize a broad spectrum of inorganic as well as organic compounds but most specifically phenolic compounds by radical catalyzed mechanism. Similarly, lignin peroxidases (LiP), heme containing proteins perform a vital part in oxidizing a wide variety of aromatic compounds with H_2_O_2_. Lignin depolymerization yields value-added compounds, the important ones are aromatics and phenols as well as certain polymers like polyurethane and carbon fibers. Thus, this review will provide a concept that biological modifications of lignin using acidophilic fungi can generate certain value added and environmentally friendly chemicals.

## 1. Introduction

The processing as well as the extraction of fossil fuels are the major prevailing environmental issues Therefore, it is the utmost need of the time to decrease the fossil fuels consumption as much as possible. The only reliable solution to this major issue is to replace the petroleum products with less costly and environmentally friendly (green) chemicals. Over 10 million tons of petrochemical materials (phenol and its derivatives) are generated annually. Thus, advancement is required to utilize new and natural raw substances for polyphenolic compounds biosynthesis [1]. Biomass is gaining much attention these days for being a renewable carbon source for chemicals, materials and energy production and hence acting as a resource to produce green products and replace fossil fuels that are decreasing day by day [2,3]. Among the plant derived raw materials, lignocellulosic biomass is most abundant and consists of three macromolecular constituents, cellulose, hemicellulose and lignin that constitutes plant cell walls. The quantities of each of these polymers are dependent on age, harvest season and plant species [4,5,6]. Identifying routes of production for both energy and value-added chemicals are imperative, and their idealistic pathways have been discussed in numerous reports [7,8].

### 1.1. Biomass Pretreatment Methods

A large proportion of lignocellulosic biomass pretreatment strategies had established that can be classified as physico-chemical, physical, chemical and biological methods [9,10]. Table 1 summarizes different pretreatment protocols for various feedstocks with their hydrolysis products, advantages and disadvantages [11].

### 1.2. Lignin

**Figure 1:**
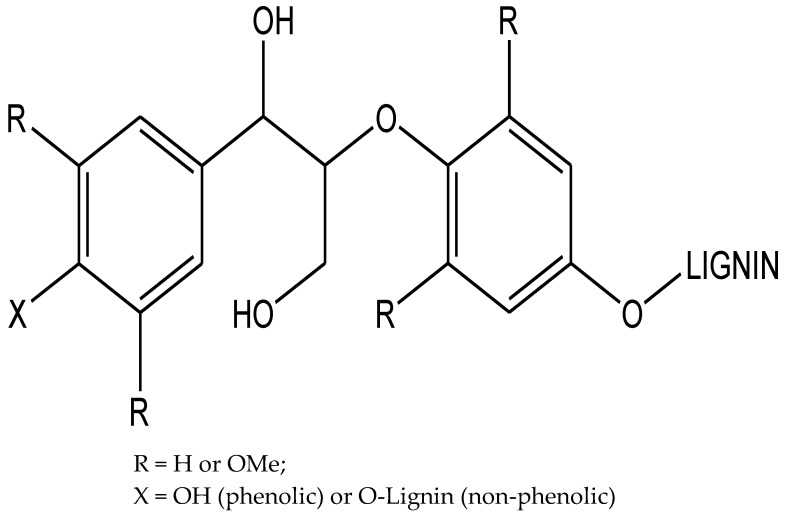
Lignin polymers can be terminated by a p-hydroxyl group or connected to additional lignin species, referred to as “phenolic” (X = OH) and “non-phenolic” (X = H or O-lignin) groups, respectively.

Lignin is primarily composed of three phenyl-propanoid monomers, namely sinapyl alcohol (S), 4-hydroxycinnamyl alcohol (H) and coniferyl alcohol (G) bonded by C-O or C-C linkages and is produced during cell wall biosynthesis by radical coupling reactions [23,24]. Plants utilize this high molecular weight, branched polymers for both water transport and defense. Around 50% of the inter-monomer linkages of lignin in most plants are the aryl ether β-O-4 bonds [23]. Lignin polymers are often terminated by a *p*-hydroxyl group, which is typically referred to as a “phenolic” group, whereas “nonphenolic” β-O-4 linkages internal to the lignin polymer can be connected to additional monomeric units (Figure 1) [25].

As the β-O-4 linkage is the most predominant in lignin, many fundamental studies have focused on understanding how this bond is cleaved in various physical and chemical environments or in the presence of homogeneous and heterogeneous catalysts [26,27]. Moreover, sophisticated NMR methods have been developed to fingerprint the presence of these aryl-ether linkages (and other linkages) in lignin to understand their fate as a function of treatment [28,29,30]. Biomass utilization efforts for both fuels and products from carbohydrates often focus on lignin removal from, or redistribution within biomass [31].

Concentrated acid hydrolysis can also be applied to depolymerize hemicellulose, cellulose, and lignin [32]. With the aim to improve carbohydrate yields from biomass, substantial efforts have also been expended to genetically modify plants to exhibit lower lignin contents or less recalcitrant lignin [33,34]. Other technologies are under development to fractionate the plant cell wall into its constituent polymers with novel solvents such as ionic liquids [35,36,37] or organic solvents (Organosolv processes) that typically utilize acid as well to depolymerize some of the lignin and hemicellulose [38,39,40].

## 2. Organic Acid Treatment

A broad scale laboratory investigations using a variety of organic solvents (ethanol, acetic acid, esters etc.) have been recognized to obtain remarkable results of both woody as well as non-woody pulping procedures [41,42,43].

### 2.1. Acetic Acid

Acetic acid, one of the first organic acids used for delignification of lignocellulosic raw material in laboratory studies, can be used as a pulping solvent uncatalyzed or catalyzed on woods [44,45]. The wood pulping properties of acetic acid is better compared with conventional chemical processes; it also possesses major benefits in contrast to other organosolv processes used at laboratory scales as reported by many researchers [46].

### 2.2. Formic Acid

Organosolv extraction is used as a substitute for delignification. Formic acid, a chemical agent for biomass fractionation, is readily available as well as a cheap organic solvent [47]. During formic acid pulping, lignin dissolves in black liquor due to cleavage of β-O-4 bonds of lignin, whereas solid cellulose remains in the residue after degradation of hemicellulose into both monosaccharides as well as oligosaccharides. Lignin precipitates out and separates out from the liquor by adding water. After pulping, formic acid can be easily recovered by distillation for reuse. Several techniques for biomass fractions in formic acid have been described including pulping in peroxy-formic acid mixtures, in aqueous formic acid and in acid-catalyzed aqueous formic acid [47,48,49,50].

Pulping of lignocellulose by formic acid is effective for delignification when formic acid concentration is higher than 80%, but delignification is inadequate at formic acid strength below 70% [47,51]. To establish potential applications of polymers resulting from formic acid pulping, the molecular characteristics and the structures of solid residues and lignin byproducts need to be characterized [52].

### 2.3. Fungal Acid Production

Low molecular weight organic acids production by filamentous fungi have attracted considerable attention due to their potential industrial applications as well as significant role in natural ecology [53,54]. Fungal natural organic acids production is thought to have many key roles in nature depending upon the type of fungi producing them. These roles are primarily either due to pH decrease consecutive to their secretion or due to direct interaction of organic acid with the environment [55,56]. The consecutive decrease in pH upon their secretion may give a competitive advantage to the acid-tolerant filamentous fungi. For ecto-mycorrhizal fungi, this decrease in pH also has been suggested to solubilize soil minerals thus releasing nutrient ions for plants and microorganisms uptake, enhancing mineral weathering [54]. For wood-decaying and saprophytic fungi, this pH acidification, caused by oxalic acid production, leads to an acid-catalyzed hydrolysis of holocellulose [57,58,59]. For this reason, *Basidiomycota* have been extensively studied for their ability to produce oxalic acid [60,61,62,63]. To better understand their role in the ecosystem; these studies have focused on both fungus and plant symbiosis or often growth on complex substrates [60,64]. In addressing the demand for sustainable alternatives to fossil fuels as energy source and chemicals, synthetic biology focuses on understanding how biological systems work and how to use them for the welfare of society. Organic acids can have multiple industrial applications as pharmaceutical, cosmetic excipients and food additives. They are fully degradable molecules and can be used as chemical intermediates or as synthons for biodegradable polymers synthesis; hence potentially replacing petroleum-based or synthetic chemicals [65].

A variety of useful organic acids are produced by fungi; citric, gluconic, malic and itaconic acids are synthesized by *Aspergillus* genera while lactic and fumaric acids are formed by *Rhizopus* genera. Large scale bio-processes can be used for certain organic acids like citric acid having the potential of fungi as organic acid production platforms [53,66]. Several acidophilic fungi are listed in Table 2 [67].

Lignin inhibits both enzymatic and microbial attack as it is the chief constituent of plant tissue’s mechanical support. By forming stable lignin-carbohydrate complexes (LCCs) with polysaccharides, lignin restricts the ruminal degradation and digestion of both cellulose and hemicellulose [68]. Some microorganisms, such as white-rot fungi and actinomycetes, can degrade LCCs [69,70], but depolymerization and subsequent metabolism of lignin seems unlikely under anaerobic conditions such as those in the rumen, because oxygen is thought to be essential for lignin breakdown [69,71,72]. Gaillard and Richards [73] found soluble compounds in the rumen that had the same ultraviolet and infrared spectra as lignin, and estimated that such soluble LCCs could correspond to as much as 40% of the total lignin intake. These soluble compounds might not be a direct result of the disruption of LCCs, but might originate instead from the microbial hydrolysis of surrounding structural polysaccharides on LCCs surfaces. On the other hand, direct degradation of lignin model compounds by ruminal microbes as well as the proposed pathways for their breakdown based on HPLC analysis of the end products were also reported [74,75,76]. Synthetic model compounds have been used to define the effect of the specific lignin binding structure on the degradability of plant cell walls. 4-Methylumbelliferone (4-MUF) is an analogue of lignin that fluoresces in free state. This compound could be very useful if it were incorporated into a lignin structure by a definite mode of linkage, since cleavage of the linkage could be detected by fluorescence [77].

Fungi can primarily degrade lignocellulosic biomass. However, augmenting the microbial activities is an array of soil macro-invertebrates, whose effects may range from simple comminution and dispersion of plant material to actual dissimilation of the structural polymer of lignocellulose [78,79]. Termites, being the most abundant and important of these invertebrates with their associated microbial symbionts have the capability to dissimilate a significant proportion of both cellulose (74–99%) and hemicellulose (65–87%) constituents of the ingested lignocellulosic plant material [80,81].

## 3. Linkages in Lignin

Lignin molecule possesses a variety of structurally correlated phenylpropanoid subunits having either C-C bonds or ether linkages known as core lignin [82]. The different linkages type in both softwood lignin and hardwood lignin together with the functional groups and their approximate proportions commonly present in a lignin macromolecule are listed in Table 3 and Table 4 [83,84]. 

The major linkage in lignin is a phenylglycerol-β-aryl ether (e.g., ring 1→14), trailed by phenylcoumaran (ring→2), diary propane (ring→11), and biphenyl (ring→5) linkages. But diphenyl ethers (ring 12→13) and pinoresinol linkages (ring 5→6) are characteristically less common [85]. The breakdown of all these linkages by hydrolysis is difficult or not possible. The basis for the complicated, nonrepetitive structure of lignin lies in its biogenesis [86].

In 1951 Freudenberg and coworkers showed that the dehydrogenative polymerization of coniferyl alcohol yielded a high molecular weight dehydrogenation polymerizate (DHP) that closely resembled spruce lignin [87,88]. Further studies revealed the explanation of a complex reaction sequence that usually takes place in plant cell walls lignifications [82,85,89,90]. The production of majority of phenoxy radicals using extracellular peroxidases starts a new cycle of non-enzymatic polymerization reactions to form oligolignols being condensed further in parallel reactions by initiating from basic identical monomers (coniferyl, sinapyl, and *p*-coumaryl alcohol). A three-dimensional complex network of non-identical oligolignols constitutes the major final product, lignin. Lignin from dissimilar phylogeny has remarkable structural differences [85].

**Figure 2:**
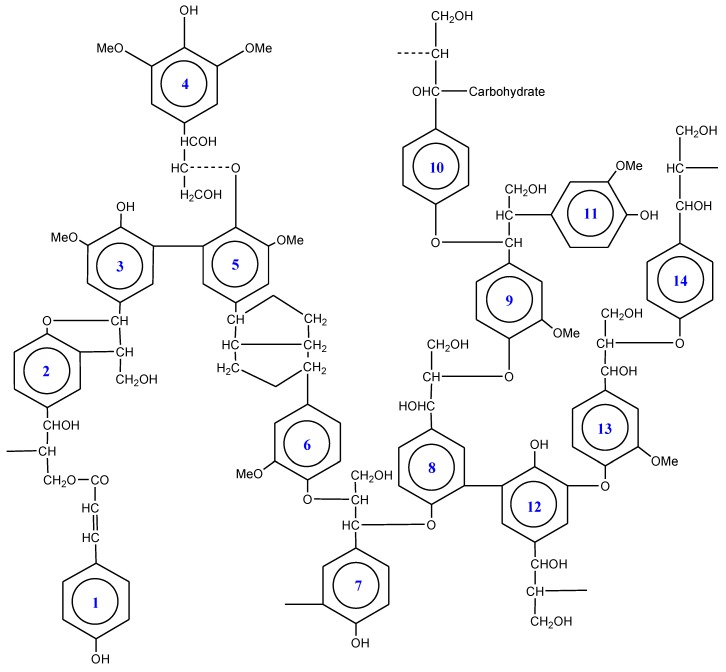
Ideal lignin molecule showing different linkages.

Softwood lignin possesses guaiacyl propane subunits (e.g., ring 13), being polymerizates of coniferyl alcohol monomers. Conversely, a mixture of sinapyl and coniferyl alcohol starts hardwood lignification that yields a characteristic mixture of syringyl and guaiacyl propane subunits (ring 4). Comparable to both types described, the grass lignin showed the greatest complication having 4-hydroxyphenylpropane subunits (ring 14). However, most grass lignin coupled with hardwoods have considerable percentage of chemically less recalcitrant linkages (5–10%) as aromatic acids being esterified to core lignin (ring 1→2) normally residing the primary hydroxyl groups at propyl side chains. Being covalently bonded with hemicellulose and possess carbohydrate polymer linkage, it is impossible to depolymerize lignin from lignocelluloses prior to partial denaturation. During the polymerization process in plant cell walls, many ethers and esters are formed by covalent linkages when several intermediates not only react with other oligolignols but also with glucuronic acids in hemicelluloses possessing both hydroxyl and carboxyl groups (Figure 2, ring 10) [85,86,89].

### Symbiotic Fungi

A subfamily Macrotermitinae, having higher termites, plays a remarkable role by forming a fascinating symbiotic association with external basidiomycete fungi belonging to genus *Termitomyces* that are being cultured in greyish-brown convoluted dynamic combs. The fungal mycelium that fills these combs have plant materials being partially digested by fungus and develops mycotetes (round white nodules) consisting of many conidia (asexual spores). Plant material gets heavy with impregnation of fresh termite faeces that ultimately becomes permeated with *Termitomyces* spp. to develop new combs. Termites can easily utilize the older or more seasoned parts of the comb together with the fungal nodules. In 1989, researchers reviewed both biology and importance of this remarkable link that proved to be a key question for fungus role in termite nutrition [91]. Evidence suggests that *Termitomyces* spp. causes incomplete digestion of both plant polysaccharides and lignin within the comb [92,93].

## 4. Lignin Degradation

Depolymerization and aromatic ring cleavage are the key steps in lignin degradation. Certain steps are involved in oxidation of lignin due to extracellular fungal enzymes:

1. Β-O-4 linkages are oxidized to arylglycerol compounds;
2. Aromatic rings are cleaved that usually follows the β-ketoadipate pathway;
3. Cleaved aromatic rings coupled with β-O-4 oxidation leads to the formation of cyclic carbonate structures [94].

### 4.1. Enzymatic Depolymerization

**Figure 3:**
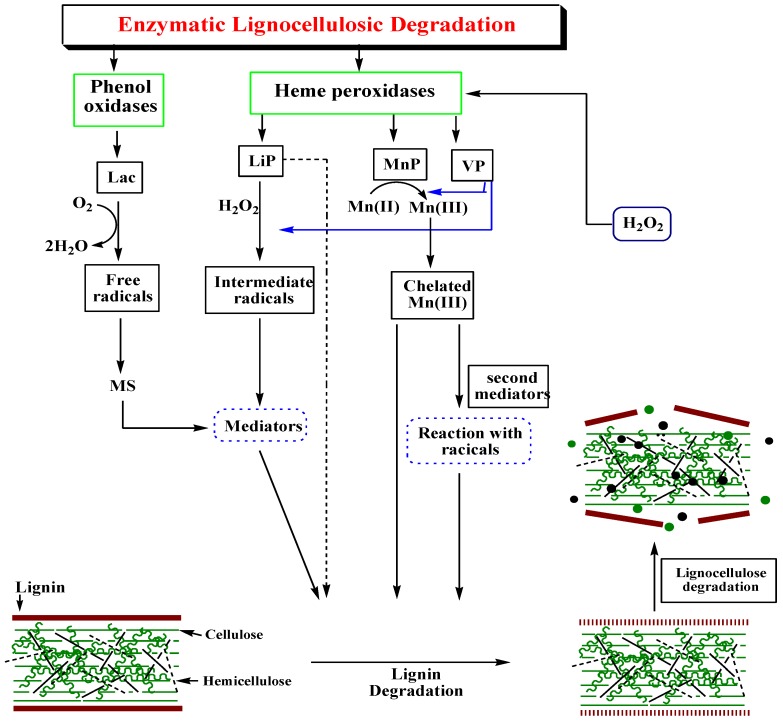
Schematic representation of the lignin degradation steps and enzymes involved.

Ligninolytic enzymes that perform the conversion of lignosulphonate considered to be the main lignin degrading enzymes [95]. Enzymatic conversion of lignocellulosic is beneficial over other physiochemical processes because of enzymatic specificity in reactions. There has been an expanding literature focusing on the ligninolytic enzymes after their discovery from white rot fungi [96]. This method is a significant alternative to the other methods due to high product yield and lower environmental impact. White rot fungi produce main lignin-degrading enzymes including heme-containing lignin peroxidases (LiP), manganese peroxidase (MnP), versatile peroxidase (VP) and copper containing laccases (benzenediol: oxidoreductase) (Figure 3) [95].

#### 4.1.1. Laccase

Laccases, being the core of interest since 19th century are one of the oldest enzymes obtained from Japanese tree, *Rhus vernicifera* as first extracted by Yoshida in 1883 [97]. For the first time in 1896, it was considered to be a fungal enzyme as demonstrated by Bertrand and Laborde [98]. These are the copper (Cu) containing proteins that contribute to oxidize a broad spectrum of inorganic as well as organic compounds but most specifically phenolic compounds by radical catalyzed mechanism [99].

**Figure 4:**
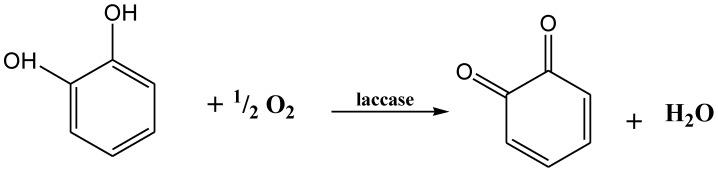
Oxygen (O_2_) reduction into water (H_2_O) by laccase.

The production of enzymes has been improved by some specific compounds which act as protein synthesis inducers. The manufacturing of recombinant laccases at industrial level has been increased by the recent success in cellular engineering and fungal molecular technology. Laccases are relatively more stable because they do not use hydrogen peroxidases (H_2_O_2_) as a cofactor. They can produce water by reducing the molecular oxygen in the presence of substrate (Figure 4) [100].

Laccases are multi-copper proteins that are characterized by their electron paramagnetic resonance (EPR) spectrum in three distinctive types:

▪ Type-1 copper: attach to two amino acids (cysteine and methionine) and two histidine ligands, because of these enzymes show blue color.
▪ Type-2 copper: attach via water and two histidine ligands.
▪ Type-3 copper: contain two copper ions each of which attach to three histidine ligands.

**Figure 5:**
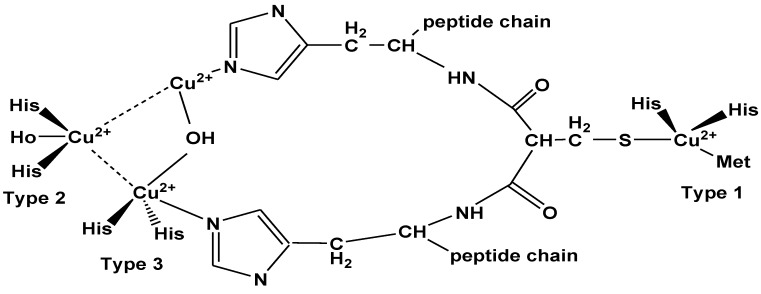
Schematic diagram of laccase active site; containing four copper which belong to type-1, type-2 and type-3 binuclear copper site based on their electron paramagnetic resonance (EPR).

Catalytic activity of laccases is performed both by type-2 and type-3 which form a trinuclear cluster (Figure 5) [101,102].

**Figure 6:**
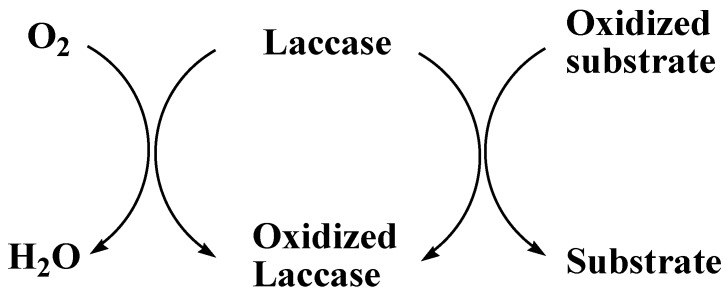
Schematic representation of redox cycles for oxidation of substrates catalyzed by laccase.

The catalytic activity is generally dependent on three binding sites with these four types of copper ions. Type-1 copper is the main primary electron acceptor and then electron transferred to the tri-nuclear cluster. The oxygen reduction into water also takes place on these binding sites. Laccases remove solely one electron to oxidize its substrate and laccase with its total reduced state contain four electrons consequently electrons gain by oxygen yielding water [103]. Substrate spontaneously forms free radical or a new compound after the removal of proton (Figure 6) [102].

An extensive amount of literature has examined the source of Laccases from fungi and plants. Its activity was also seen in bacteria viz. *Streptomyces griseus*, *Azospirillum lipoferum*, *Marinomonas mediterranea*, and *Bacillus subtilis* [104,105,106]. There are abundant types of fungi that show Laccases activity including *Neurospora crassa*, *Pyricularia bryzae*, *Pleurotus*, *Pholiata*, *Polyporus versicolor* A, B, and *Aspergillus nidulans*. However, researchers show much interest in basidiomycetes like *Agaricus bisporus*, *Lentimus edodes*, *Trametes versicolor* and *Pleurotus ostreatus* since they produce laccases that are involved in lignin degradation [107]. Laccases from *Trametes versicolor* (LTV) and *Agaricus bisporus* (LAB) are easily available commercially and have various applications in different fields including pulp and paper industry, textiles, environmental aspects, the food processing units, pharmaceutical business and nano-biotechnology [102].

Additionally, voluminous literature covers the LAB and LTV regarding their reactions and production [108]. Laccases synthesized specially from white rot fungus (LAB and LTV) can cause lignin degradation due to their ability to further rearrange the phenoxy radical by C_α_-C_β_ cleavage as well as the benzyl hydroxyls oxidation. Lignin polymer is too large to penetrate active site of laccase so it could not oxidize directly by laccase. Furthermore, a mediator; an additional compound is required to deal with this limitation [109].

#### 4.1.2. Laccase-Mediator System (LMS)

For the depolymerization of lignin, laccases require a mediating agent known as intermediary substance or mediator. Mostly laccase mediators are low molecular weight and aromatic compounds. The combination of laccases with mediators increase the yields and rates in conversion of laccase-substrate as well as it adds new reactions to substrate without which enzyme shows no or just marginal activity. Consequently, LMS enhances the range of substrate to oxidize compounds with higher redox potential (E°) compared to laccases (LMS E° lies above +1100 mV but laccase allows to oxidize molecule in limited range of +475 to +790 mV) [110].

**Figure 7:**
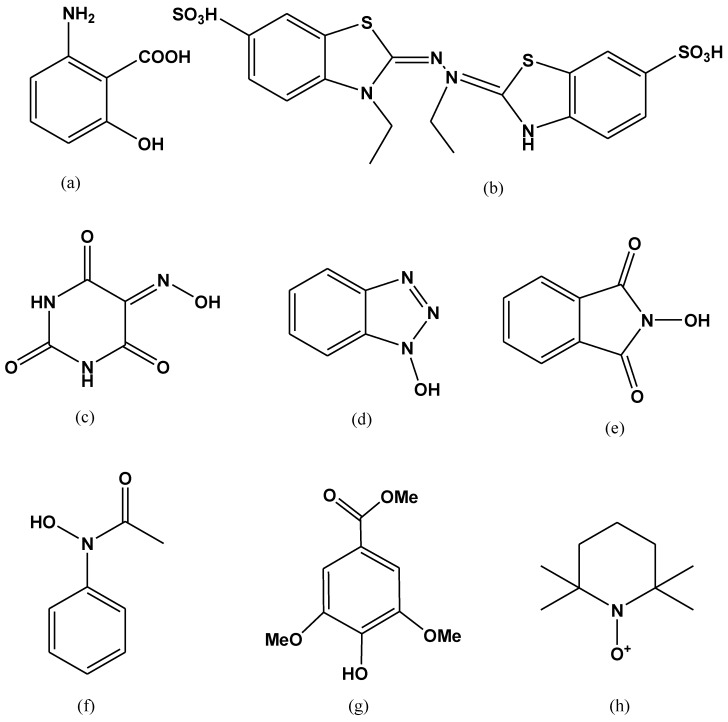
Examples of laccases mediators. (**a**) 3-Hydroxyanthranilic acid (HAA); (**b**) 2,20-azino-bis-(3 ethylbenzothiazoline-6-sulphonic acid) (ABTS); (**c**) *N*-hydroxybenzotriazole (HBT); (**d**) *N* hydroxyphtaimide (HPI); (**e**) violuric acid (VLA); (**f**) *N*-hydroxyacetanilide (NHA); (**g**) methyl ester of 4 hydroxy-3,5-dimethoxy-benzoic acid (syringic acid); (**h**) 2,2,6,6-tetramethylpiperidine-1-yloxy (TEMPO).

Numerous artificial mediators have been discovered oxidizing the non-phenolic structural moieties of lignin [111]. They remain the subject of wide range of study, from the very first described laccase-mediator, ABTS; to the synthetic mediators of -NOH- type (e.g., 1-hydroxybenzotriazole (HBT), *N*-hydroxyphtalimide (HPI), violuric acid (VLA), *N*-hydroxyacetanilide (HAA) and *N*-hydroxyacetanilide (NHA)) and the stable one 2,2,6,6-tetramethyl-1-piperidinyloxy free radical or TEMPO [111,112,113]. ABTS has been considered the best substrate-mediator laccase. It speeds up the rate of reaction by moving the electron towards electron accepting compounds from the donor substrate. Two stages are involved in the oxidation of ABTS. In the earlier stage, fast oxidation occurs and cation radical (ABTS^+^) is formed, after that di-cation (ABTS^2+^) formed by the slow oxidation of cation radical (Figure 7) [114].

**Figure 8:**
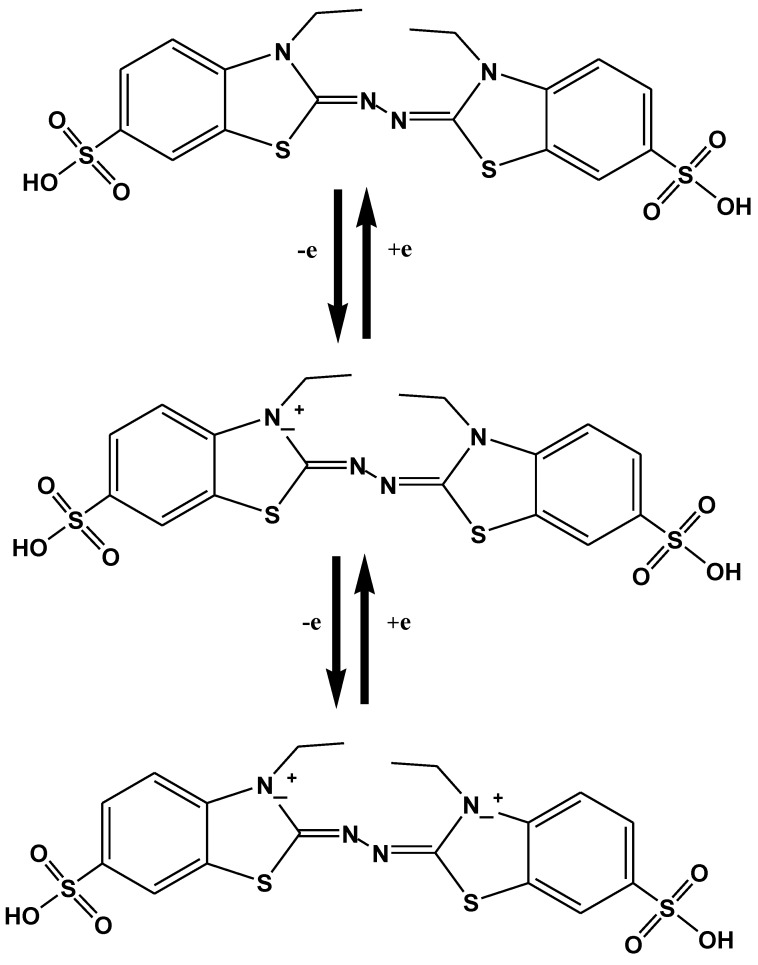
Oxidation of ABTS catalyzed by laccase.

A large body of literature has explained ABTS application of lignin degradation using laccase. The use of mediators, most probably ABTS is unique for the oxidation of lignin subunits. Many workers examined the Kraft lignin oxidation by *Trametes versicolor* (LTV) laccase and stated that ABTS coupled with laccase enhance the catalytic activity of laccase to generate lignin subunits having an average weight of 5300 g/mol [112]. The mechanism of ABTS oxidation indicates that ABTS^2+^ di-cation only act as an intermediate, for oxidation of non-phenolic structures. Conversely, ABTS^+^-cation radical accounts for phenolic structures [114]. In previous studies, researchers mostly concentrated on the oxidation mechanism of ethers, alcohols and lignin model compounds. Extensive research has described the effects of mediators and laccase enzyme on lignin model compounds to fully recognize the laccase reaction owing to the lignin structure complexity [112] (Figure 8).

#### 4.1.3. Model Compounds of Lignin

The structural variability and complexity of lignin provoked the use of various lignin model compounds in its place to study the lignin depolymerization [115]. Such model compounds bear a resemblance to lignin polymer and investigation of their reactivity gives understanding about the reactivity of lignin polymer itself. Several factors lead to the use of lignin model compounds:

1. to perceive the interaction between lignin and enzymes by using lignin model compounds in place of lignin due to their simple structure;
2. many model compounds contain lignin-related linkages i.e., β-O-4, α-O-4, β-5, 4-O-5, etc. so their reactivity give the information relevant to lignin-enzyme interaction;
3. the product and analysis of such model compounds are relatively easy as compared to lignin. Many publications give the idea about the interaction of lignin with laccase; though, the lignin degradation mechanism is much more difficult to understand [116].

#### 4.1.4. Lignin Peroxidase (LiP)

Lignin peroxidases are heme containing proteins having an iron protoporphyrin prosthetic group, first isolated from *Phanerochaete chrysosporium*. These peroxidases catalyze the oxidation of a broad variety of aromatic compounds in the presence of H_2_O_2_ [117].

This enzyme had been completely characterized and its catalytic mechanism was studied previously in oxidizing substrate. Firstly, the enzyme is oxidized using hydrogen peroxide (H_2_O_2_) to LiPI (intermediate of LiP) and water. LiPI then converts to LiPII and substrate radical (VA^+^) by the oxidation of first molecule of veratryl-alcohol (VA). LiPII use the second veratryl alcohol (VA) by the reduction of the substrate and the enzyme recover in its original form as mentioned in Equation (1) [118].

(1)           **Enzyme (LiP) + H_2_0_2_→ LiP I + H_2_0** **LiP I + VA → LiP II + VA^+^**               **LiP II + VA → Enzyme (LiP) + VA^+^**

Since 1986, veratryl alcohol (VA) had been a redox mediator for LiP; it did not react with lignin in the absence of veratryl alcohol. So, lignin depolymerization via LiP was performed by adding veratryl alcohol [119].

#### 4.1.5. Manganese Peroxidase (MnP)

Manganese (Mn) is required for MnP synthesis. This enzyme has the pivotal role for earlier stages of degrading lignin polymer [120] and are produced by wide-ranging species of white rot basidiomycetes like *Phanerochaete chryosporium* [121]. For last 25 years, production of heme-peroxidases remained an interesting subject for researchers which include both manganese peroxidase (MnP) and lignin peroxidase (LiP) [122]. MnP, like LiP are heme containing proteins as well that use H_2_O_2_ as a co-substrate in the substrate oxidation. Like LiP, MnP also produces the intermediates (MnP-I and MnP-II) in its catalytic cycle [118].

**Figure 9:**
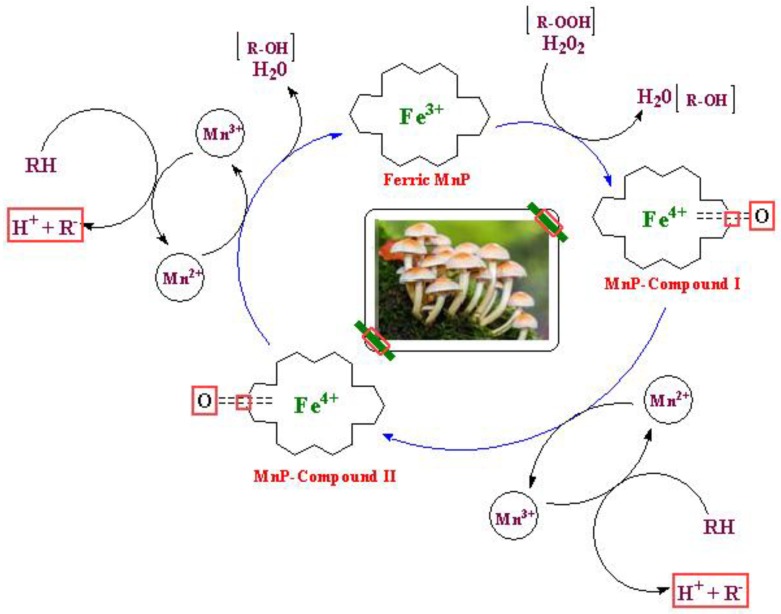
Schematic representation of manganese peroxidase (MnP) catalyzed redox cycles for Mn^2+^.

The nature of substrate makes the main difference between MnP and LiP. Unlike LiP, primary substrate of MnP is Mn(II) instead of phenol and produces Mn(III) which is highly reactive and oxidizes a variety of phenolic compounds. Firstly, iron-peroxide complex is formed when native ferric MnP bound to H_2_O_2_. MnP-Compound-I along with a molecule of water produces by the transfer of electrons from MnP. Mn^2+^ oxidized to Mn^3+^ and transfer the electron to the porphyrin intermediate while MnP-Compound-I transformed to MnP-compound-II [123]. MnP-II reduces in a similar way and regenerate the native MnP along with a second water molecule. Mn^3+^ ion chelated with organic acids (malonate and lactate) makes possible Mn^3+^ release from the active site of MnP. This detachment increases the oxidation rate by stimulating the MnP activity. Chelates of Mn^3+^ ion cause the oxidation of many substrates or the removal of radicals (Figure 9) [124].

#### 4.1.6. Versatile Peroxidases (VP)

A novel peroxidase from *Pleurotus eryngii* was reported and this peroxidase contains both main peroxidase properties (LiP and MnP) that can modify lignin molecule without the involvement of external mediator [125]. This enzyme, named as versatile peroxidase (VP), indicates that it has properties of both LiP and MnP and can oxidize various substrates including Mn^2+^, phenolic compounds and non-phenolic aromatic compounds e.g., veratryl alcohol [126]. Versatile peroxidase is isolated from white-rot fungi types like *Bjerkandera* spp*.* strain BOS55, *Pleurotus ostreatus* and *Bjerkandera adusta* [127]. Furthermore, VP are characterized by having extensive specificity for aromatic substrates, making them highly beneficial in certain applications including recalcitrant pollutants bioremediation as well as gaining major industrial interests these days [128].

In summary, heme containing peroxidases (LiP, MnP and VP) also have some drawbacks that limit their use as well. MnP, LiP and VP involve the use of H_2_O_2_ for their catalytic activity while laccases only require O_2_ which they absorb from the atmosphere directly. Peroxidases are extreme expensive and are not commercially available yet, in contrast, laccases are available at low prices. In comparison to peroxidases, laccases offer selection of mediator compounds for the process requirements. Consequently, laccase is a potential enzyme for degradation of lignin with promising applications that might improve efficiency and productivity with low investment cost [129]. Fungal enzymes biosynthesis for the depolymerization of lignin on industrial scale or chemical functionalization has been hindered by complications like culturing white rot fungi on an industrial scale as well as in lignin-degrading enzymes expression in other fungi [130,131].

## 5. Bonds Cleavage in Lignin

Variety of depolymerization protocols are employed to yield ‘green’ chemicals from lignin. The production of aromatic chemicals might be achieved through several processing routes using the lignin enriched fractions [132]. The regulated breaking of different linkages in lignin requires detailed information regarding the stability of the bonds under different conditions in addition to understand the lignin decomposition mechanism. In lignin, both ester and ether bonds are easily hydrolysable. Lignin can also be degraded by means of biological methods with micro-organisms, by chemical routes or via sun light (UV) [133].

### Monomeric Lignin Molecules

Selective depolymerization involving C-O and C-C bond rupturing produce an excess of complex aromatics structures that are either difficult to generate via conventional petrochemical ways. These compounds are correlated to the fundamental building blocks of lignin and are highly desirable due to their production in a reasonable commercial amount. However, two barriers would have to be overcome. The first one is the advancement in technology for careful bond-scission to separate out the monomeric lignin structures, although this technology would be more difficult to develop than the other destructive processes that yield phenols or BTX. Secondly, applications and markets for lignin monomers are needed to be developed. For these reasons, this technology has long-term applications and currently their large-scale use is unknown [134].

## 6. Green Chemicals

By exploring the chemical worth of biomass, green chemical technologies developed to capture the resources and maximize the production of value added plus environmental friendly chemicals. In this integrating approach, high value chemicals co-produce which maximizes the use of all biomass components, waste streams and by-products virtually with keeping environmental footprint low [105]. Green chemicals obtained from the lignin are linked to the well-being of the environment with the potential production of renewable fuels, polymer building blocks and aromatic monomers such as phenol, vanillin, benzene, toluene, and xylene (BTX) [135].

### 6.1. Lignin for Production of Aromatic Chemicals

**Figure 10:**
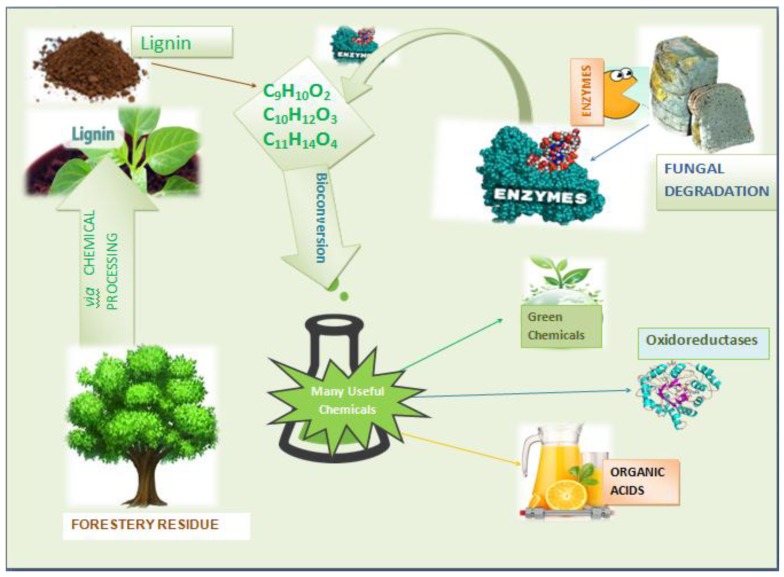
Commercial Lignin Transformations.

Lignin (the renewable raw material) is probably present in ample amounts for the synthesis of aromatic substances at industrial level. It seems easy to conclude that efficient and direct conversion of lignin into low molecular weight and distinct aromatic compounds is highly remarkable goal. But the synthesis of defined high-volume aromatic chemicals using diverse and physically intricate lignin is feasible and long-term opportunity, although it is a most challenging goal to achieve (Figure 10) [136].

#### Aromatics/Phenolics

Benzene, toluene and xylene (BTX) have large-scale of applications. Therefore, it has great potential in chemical industry. Lignin-based BTX is similar to BTX from petroleum and so can be used as a replacement for it. BTX represents 60% of all aromatics on market and 24% of the global petrochemical market [137].

Lignin can be depolymerized into various aromatic components. As these compounds are obtained from lignin, the first and the foremost duty is to eradicate the oxygen containing functional groups by decarboxylation, decarbonylation, dehydroxylation and demethoxylation [138]. Benzene is a resourceful petrochemical building block from which more than 250 products could be formed. Cyclohexane, ethyl benzene and cumene are the chief derivatives. The xylenes product well-known as mixed xylene contains four different isomers: ortho-xylene, para-xylene, meta-xylene and ethyl benzene. Toluene is gaining importance for the xylenes manufacturing through disproportionation of toluene and trans-alkylation with C-9 aromatics [139]. Aromatic complexes are found in several different configurations. However, most modern complexes of aromatics are considered to maximize the yield of para-xylene, benzene and sometimes ortho-xylene [140].

The main advantage of generating phenols from lignin is that nowadays phenol prices are quite high based on prices of oil. On the other hands, lignin as part of renewable source has relatively stable market value [137]. By focusing on phenol and its derivatives, the phenolic hydroxyl and the aromatic ring needs to be remain intact and thus less energy will be required to convert polyphenolic ligneous complex into useful compounds [141].

### 6.2. Lignin Valorization to Polymers

Advances in fractionation, catalyst development and purification technologies are necessary to obtain the required final depolymerized lignin by-product [142]. Shortly, lignin has the capability to substitute polymers such as polyacrylonitrile (PAN) to manufacture carbon fiber [143].

#### 6.2.1. Carbon Fiber

Carbon fibers with properties like low density, high stiffness and extensive strength are highly valuable composite material [144]. They have wide range of applications that are increasing day by day. The prices of carbon fibers for automotive industry is relatively high these days. The precursor for carbon fiber is polyacrylonitrile (PAN) that makes 50% of all production costs. Lignin plays its role to lessen this production cost. Lignin, being a replacement for PAN makes the process much cost effective with potential usage involve manufacturing of sport goods and aircrafts, utilization in automobile industry as well as in civil engineering [137].

Kayacarbon developed the first lignin based carbon fibers, while Nippon Kayaku Co. in Tokyo, Japan made its commercial availability possible. Initially, lignin is melt-spun at high rates to generate economical lignin-derived carbon fiber that demands high purity lignin. The contaminations like polysaccharides, salts, water and further volatiles should be eliminated to obtain required results. Both Graf Tech International Holdings and Oak Ridge National Laboratory hold the record of generating elevated temperature thermal insulation prototypes by using lignin based carbon fibers [145].

#### 6.2.2. Polymer Blends

Unmodified lignin does not have properties to be used as material. On the other hand, it can be blended with other synthetic or bio-based polymers. Lignin usually acts like UV degradation stabilizer or thermo-oxidation stabilizer. This functions lignin fulfils if it is blended with polyethylene, polystyrene, polypropylene or natural rubber [144].

#### 6.2.3. Binders

Lignin do have major impact in the agrochemicals sector as well. Many applications including resins and foams sectors coupled with polymers and cement, which are tremendous dispersing agents and binders with dust controlling abilities are the results of lignosulfonates. Concrete industry is the main and the largest applications of lignosulfonates [146]. The strong dispersing agent properties of lignosulfonates allow less water consumption that provide the resulting concrete with better durability, elevated density and higher compressive strength etc. before utilizing as workable mixtures [147].

#### 6.2.4. Polyurethane

Polyurethane, with an extensive variety of products in varied sectors, like paints, foam, adhesives, elastomer etc. is the most versatile polymers obtained by lignin valorization. Through its great insulation and mechanical properties, Rigid polyurethane (RPU having high mechanical and insulator capabilities) coupled with foams and elastomers is frequently used in freeze sectors, equipment manufacturing, automotive industry and construction in addition to nautical applications [148].

### 6.3. Certain New Products from Lignin Valorization

Several academic groups are working hard to attain a vast scope of new applications and useful products including fuel cells and high-performance materials, composites and batteries from lignin along with the classic aromatics and polymers [143,149,150]. Furthermore, by direct lignin fuel cells, lignin can also be used as fuel [151]. A novel N-doped fused carbon fibrous mat constructed via 9:1 combination of lignin:polyethylene oxide has been reported [152]. Since lignin possesses an aromatic character making it a remarkable preliminary material for graphite electrodes [153]. This can also prove to be an important and useful source for both fuel cells and lithium batteries as described presently [149].

## 7. Conclusions

More recent research work including the biological modifications of lignin indicates that lignin can be depolymerized into variety of useful chemicals of industrial importance. For the progress of an economical viable lignin valorization path to synthesize aromatic chemicals, advanced methods are required to assess the ideal conditions, appropriate hydrogen donors together with bio-refinery catalysts. There is also much need of the time to further develop this process for the commercial production of high purity lignin and lignin based byproducts.

In nature, lignocellulosic residues obtained via municipal solid wastes, agricultural source, grass, wood and forestry substances are available in bulk quantities and have an enormous bio-conversion potential. As a renewable resource, they are an important source of both biologically and chemically useful products. Lignin, when accumulated in sufficient amounts at places where agricultural residues reveal a discarding nuisance result in environmental decline coupled with valuable materials loss that can be helpful in paper and pulp industry as well as biomass fuel production and composting.

Varieties of innovative markets for lignocellulosic residues especially of lignin like Benzene, Toluene and Xylene (BTX) have been identified in recent times. Low cost bioremediation projects by utilizing fungi seem to be promising as they are the source of well-organized lignocellulose depolymerization enzyme machinery. However, additional consideration of the innumerable other enzymes coupled with organic acids for depolymerization reactions and its molecular features will be desired. The most remarkable task is to assimilate various enzymes roles and organic acids together with natural lignin degradation using a variety of microbes. Thus, lignin valorization by organic acids seems to be much more effective and safe to increase the product quantities and as well as to decrease costs compared to certain other costly manufacturing protocols.

## Figures and Tables

**Table 1:** Summary of various pretreatment methods.

Pretreatment Methods	Feedstock	Sugar Yield	Advantages	Disadvantages	References
**Biological**	Softwood Wheat straw, rice straw	20–50% Reducing sugar	Low energy consumption Cost effective Moderate reaction conditions	Required large sterile area Low hydrolysis rate	[12]
**Ionic liquids (ILs)**	Agricultural residuals: wheat straw, bagasse, corn stover, peanut and poplar sawdust	60–85% Reducing sugars	Efficiently dissolution of cellulose	Great amounts of expensive ILs are needed Solutions viscous and difficult to handle	[13]
**Ozonolysis**	Agricultural residuals: wheat straw, bagasse, peanut and poplar sawdust	45–90% Reducing sugars	Moderate reaction conditions Efficient lignin degradation	Costly protocol Requires large amount of ozone	[14]
**Alkali pretreatment**	Agricultural residuals: rice straw, wheat straw, woody material, sunflower stalk and corn stover	65–85% Reducing sugars	Room temperature Destroy lignin	Less sugar degradation	[15]
**Dilute acid**	Agricultural residuals: wheat straw, and poplar sawdust	45–80% Reducing sugars	Fast and do not need acid recycling	Formation of inhibitors Require high temperature and pressure	[16]
**Concentrated acid**	Agricultural residuals: wheat straw and bagasse	60–90% Reducing sugars	High sugar conversion	Costly and need special reactors Highly corrosive and toxic	[17]
**Organosolv**	Agricultural residuals: wheat straw and sugarcane bagasse	Up to 60% of reducing sugars	Pure lignin removal as by-product Hydrolysis of lignin and hemicellulose	Costly process Requires recycling and drainage of solvents	[18]
**Steam explosion**	Agricultural residuals: wheat straw, corn stalk and sugarcane bagasse MSW Hardwood	50–70% Reducing sugars	Cost effective Less hazardous process Lignin transformation Good sugar recovery	Incomplete destruction of lignin carbohydrate matrix Inhibitor compounds generation	[19]
**Liquid hot water (LHW)**	Agricultural residuals: wheat straw, corn stover, sunflower stalks and sugarcane bagasse	80–94% Reducing sugars	Pure hemicellulose recovery No addition of catalysts High sugar recovery	High energy demand Dealing with left over solid mass	[20]
**Extrusion**	Agricultural residuals: wheat straw and rice straw	50–75% Reducing sugars	Moderate temperature Good yield Less hazardous	Partially hemicellulose degradation Generation of inhibitors	[21]
**Ammonia fiber explosion (AFEX)**	Municipal solid waste Agricultural residuals: wheat straw, baggase and rice straw	Up to 80–90% of reducing sugars	Low formation of inhibitors Efficient lignin removal Moderate process conditions	Costly No efficiency with high lignin contents	[22]

**Table 2:** Acidophilic fungal strains [67].

Compounds	Fungal Strains
***Ascomycota***	
Itaconic acid	*Aspergillus terreus*
Fumaric acid	*Aspergillus niger*
Ascorbic acid	*Aspergillus niger*
Butyric acid	*Aspergillus flavus*
Isobutyric acid	*Aspergillus niger*
Malic acid	*Aspergillus niger*
Citric acid	*Aspergillus niger*
Succinic acid	*Aspergillus flavipes*
Lactic acid	*Aspergillus niger*
Oxalic acid	*Aspergillus niger*
Formic acid	*Aspergillus flavipes*
Acetic acid	*Aspergillus niger*
Propionic acid	*Aspergillus niger*
Gluconic acid	*Aspergillus niger*
***Basidiomycota***	
Gluconic acid	*Pycnoporus coccineus*
Oxalic acid	*Ganoderma weberianum*
Formic acid	*Pycnoporus coccineus*

**Table 3:** Common linkages in lignin [83].

Linkage Type	Share in Softwood Lignin (%)	Share in Hardwood Lignin (%)
β-O-4	45–50	60
5-5	10–27	3–9
Β-5	9–12	6
α-O-4	2–8	7
β-β	2–6	3–12
β-1	7–10	1–7
4-O-5	4–8	7–9
Dibenzodioxocin	5–7	1–2

**Table 4:** Functional groups in lignin [84].

Functional Groups	Abundance per 100 C_9_-units	
	Softwood Lignin	Hardwood Lignin
Methoxyl	90–97	139–158
Phenolic hydroxyl	15–30	10–15
Carbonyl	10–20	17–24
Aliphatic hydroxyl	115–120	88–166
